# Diffusion-Weighted Imaging for Skin Pathologies of the Breast—A Feasibility Study

**DOI:** 10.3390/diagnostics14090934

**Published:** 2024-04-29

**Authors:** Dominika Skwierawska, Frederik B. Laun, Evelyn Wenkel, Lorenz A. Kapsner, Rolf Janka, Michael Uder, Sabine Ohlmeyer, Sebastian Bickelhaupt

**Affiliations:** 1 Institute of Radiology, Universitätsklinikum Erlangen, Friedrich-Alexander-Universität Erlangen-Nürnberg (FAU), Maximiliansplatz 3, 91054 Erlangen, Germany; 2 Radiologie München, Burgstraße 7, 80331 München, Germany; 3 Medical Faculty, Friedrich-Alexander-Universität Erlangen-Nürnberg (FAU), 91054 Erlangen, Germany; 4 Chair of Medical Informatics, Friedrich-Alexander-Universität Erlangen-Nürnberg (FAU), Wetterkreuz 15, 91058 Erlangen-Tennenlohe, Germany

**Keywords:** magnetic resonance imaging, diffusion-weighted imaging, skin cancer, breast cancer, apparent diffusion coefficient

## Abstract

Several breast pathologies can affect the skin, and clinical pathways might differ significantly depending on the underlying diagnosis. This study investigates the feasibility of using diffusion-weighted imaging (DWI) to differentiate skin pathologies in breast MRIs. This retrospective study included 88 female patients who underwent diagnostic breast MRI (1.5 or 3T), including DWI. Skin areas were manually segmented, and the apparent diffusion coefficients (ADCs) were compared between different pathologies: inflammatory breast cancer (IBC; *n* = 5), benign skin inflammation (BSI; *n* = 11), Paget’s disease (PD; *n* = 3), and skin-involved breast cancer (SIBC; *n* = 11). Fifty-eight women had healthy skin (H; *n* = 58). The SIBC group had a significantly lower mean ADC than the BSI and IBC groups. These differences persisted for the first-order features of the ADC (mean, median, maximum, and minimum) only between the SIBC and BSI groups. The mean ADC did not differ significantly between the BSI and IBC groups. Quantitative DWI assessments demonstrated differences between various skin-affecting pathologies, but did not distinguish clearly between all of them. More extensive studies are needed to assess the utility of quantitative DWI in supplementing the diagnostic assessment of skin pathologies in breast imaging.

## 1. Introduction

Breast magnetic resonance imaging (MRI) provides high-resolution morphological information, facilitating the detection and characterisation of suspicious lesions [1]. Among the routine sequences, diffusion-weighted imaging (DWI)—a contrast agent-free MRI technique based on the Brownian motion of water—has gained an increasingly important role. DWI measures the diffusion of water molecules in tissue, whose motion is correlated to tissue microstructure [2,3,4]. Amongst several quantitative assessment approaches, the apparent diffusion coefficient (ADC) derived from diffusion-weighted images has been reported to support lesion characterisation [5,6,7,8,9,10,11,12,13,14,15,16,17], since malignant tumours frequently demonstrate lower ADCs than benign lesions or normal tissue [18]. Therefore, DWI commonly complements multiparametric dynamic contrast-enhanced MRI protocols and has been reported to improve specificity in clinical reading [19,20,21], aiding in the reduction of false positives or indeterminate assessments. Thus, although not mandatory, DWI sequences are increasingly incorporated into multiparametric breast MRI protocols.

Pathologies in the female breast can affect or originate from the fibroglandular tissue (FGT) components and, in relatively rare cases, from the skin [22,23]. The spectrum of skin pathologies includes benign processes such as, e.g., benign skin inflammation (BSI), associated with mastitis. However, it can also indicate the presence of other pathologies of the skin, like Paget’s disease (PD), which represents 1–3% of all breast cancers and mostly occurs in postmenopausal women [24]. Secondary skin-involvement in non-specific breast cancer (SIBCs) or the primarily highly aggressive inflammatory breast cancer (IBC) are further rare pathologies, with the latter accounting for 2–4% of all breast cancers [25,26]. Symptoms associated with skin involvement in IBC can show an overlap with the spectrum of symptoms in skin affection of benign mastitis [23,27,28], presenting with symptoms such as redness, swelling, warmth, and tenderness [28]. In case of such symptoms, antibiotics and observation of success are potential initial pathways which, depending on therapeutic success, might then be followed or accompanied by the decision about a potential invasive work-up, such as a biopsy.

Whilst MR imaging can help by differentiating pathologies, there is limited data on whether MR imaging might provide specific aid for assessing skin involvement in diseases, e.g., differentiating between benign inflammation and skin infiltration in breast cancer.

DWI’s technical characteristics suggest its potential for assessing lesions in breast MRI beyond the skin tissue. It may also support the evaluation of the skin itself by providing microstructural correlates of the alterations, determining the extent of cancer spread and assessing the extent of skin thickening [18,29,30,31].

Our study aimed to gain the first insight into the capability of DWI and first-order ADC statistics to support the characterisation of breast skin pathologies. Due to the rarity of such clinical cases, this study serves as an initial exploration and evaluation of the potential of this approach.

## 2. Materials and Methods

### 2.1. Study Population

**Figure 1:**
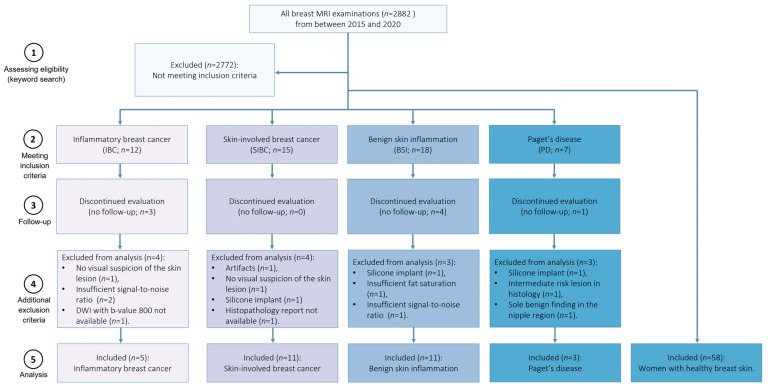
Selection of the population. Among women examined between 2015 and 2020, 30 patients with skin pathologies met the inclusion and exclusion criteria. Additionally, fifty-eight women with healthy skin (*n* = 58) have been included.

This institutional review board-approved retrospective study included breast MRI examinations performed at the University Hospital Erlangen (Germany) between 2015 and 2020. All breast MRI examinations were conducted as part of the clinical routine. This cohort was part of previously published works by Liebert et al. [32] and Kapsner et al. [33,34], in which image quality assessment and artifact detection were investigated. The inclusion criteria were female patients with clinically indicated (e.g., preoperative or postoperative evaluations, assessment of multifocal disease or unclear findings, screening in cases of elevated breast cancer risk) breast MRI examination. The MRI protocol consisted of multiparametric imaging, including multi-b-value DWI. Further, the examinations were stratified into five subgroups based on the clinical reports regarding the presence of one of the considered diseases: Paget’s disease, skin-involved breast cancer of no special type, inflammatory breast cancer, or benign skin inflammation. The BSI category comprised mastitis, chronic inflammation, and lymphedema. Identifying patients eligible for inclusion in this study involved a keyword search of our in-house structured database (see Supplementary Materials—text document Supplemental Digital Content 1: Keyword List, which contains the keywords used for patient identification). It included terms characteristic of breast skin pathologies, such as “benign skin inflammation”, “Paget”, “skin”, “thickened skin”, “flush”, “redness”, and “oedematous”. Since the medical reports were in German, the keywords were chosen accordingly. Fifty-eight women with healthy breast skin (i.e., patients with a Breast Imaging Data and Reporting System [BI-RADS] 1 or BI-RADS 2 assessment) were included. Besides these 58 cases, the search identified 52 patients with target findings for inclusion. The following exclusion criteria were applied: image artifacts affecting diagnostic image quality (e.g., insufficient fat saturation in DWI data; *n* = 2), the presence of silicone implants within the breast (*n* = 3), a lack of histology results with only radiologic suspicion (*n* = 9), intermediate risk lesion in histology (*n* = 1), no visual suspicion of the skin lesion (*n* = 2), missing DWI images (*n* = 1), and insufficient signal-to-noise ratio (SNR; *n* = 3). Furthermore, one case initially assigned to the Paget’s disease group had a histologically confirmed benign finding. Nevertheless, we opted to exclude the case because it was the sole benign finding in the nipple region. Figure 1 presents the total number of breast MRI examinations performed within the 2015–2000 period, as well as the exclusion processes of patients. The reference was either the radiologic report (e.g., health skin cohort) or/and the histopathological reporting in the radiology information system.

### 2.2. MRI Protocol

Since this study was retrospective, patient examinations were conducted with multiple scanners and sequence settings reflecting the clinical routine examination process. MRI examinations were performed using either a 1.5 Tesla (Avanto [*n* = 55] or Aera [*n* = 8]; Siemens Healthineers, Erlangen, Germany) or 3 Tesla (Skyra Fit [*n* = 13] or Magnetom Vida [*n* = 12]; Siemens Healthineers, Erlangen, Germany) system using a dedicated multi-channel breast coil. The multiparametric breast MRI protocol included a T_1_-weighted sequence before and dynamically acquired after the injection of gadolinium-based contrast agents, a T_2_-weighted sequence, and a diffusion-weighted sequence acquired in axial orientation with echo-planar readout and b-values of 50 s/mm^2^, 750 or 800 s/mm^2^, and sometimes 400 and 1500 s/mm^2^. Since our study focuses on quantitative DWI assessment, the imaging parameters for the diffusion-weighted sequences are detailed in Table 1.

### 2.3. Data Processing

All imaging data relevant to this study were transferred from the routine clinical Picture Archiving and Communication System (PACS) to a dedicated research workstation. Then, manual segmentations were performed by a physicist (D.S., one year of experience in breast DWI) supervised by a board-certified radiologist controlling all segmentations in consensus (S.B., nine years of experience in breast DWI). Skin pathologies were identified per the radiologists’ report using all available multiparametric breast MRI protocol sequences.

Then, segmentations were conducted as follows: The segmentations were defined manually on diffusion-weighted images (b-value 750–800 s/mm^2^) using the Medical Imaging Interaction Toolkit (MITK; v2018.04; German Cancer Research Center (DKFZ), Heidelberg, Germany). The segmentations were contoured carefully, avoiding necrotic parts of the lesion, major artifacts (e.g., due to folding skin), and cystic or calcified structures within the affected breast region. The boundaries were specified to be smaller than the actual visual volume of the affected area of the skin to minimise partial volume effects (a rim of roughly one voxel was kept), which is particularly important in the skin. While the segmentations did not always cover the entire volume of the lesions or area affected by tissue alterations, they commonly included several slices, so in most cases, the volume of interest (VOI) included in this study was three-dimensional. In cases with multiple skin pathologies (uni- or bi-lateral), only one VOI per patient was evaluated and included in this study. One representative slice of the breast MRI examination, with a clearly visible healthy skin region, was selected for the control group, and segmentations were performed by visually delineating skin regions (mean nr of voxels = 29). In the healthy control group, the delineation process was performed using the b = 50 s/mm^2^ images, since the visibility of the skin often decreased rapidly at higher b-values. Segmentation masks were stored as .nii files and further processed as described below.

### 2.4. ADC Calculation

The ADC was calculated using two methods. The first method involved calculating the diffusion coefficient using the mean signal value, $\bar{S}\left(b\right)$, within the VOI:

(1) $$ADC=\frac{ln(\frac{\bar{S}\left(b_{2}\right)}{\bar{S}\left(b_{1}\right)})}{b_{1}-b_{2}},$$

where $\bar{S}\left(b_{1}\right)$ and $\bar{S}\left(b_{2}\right)$ are the mean signal intensities of the VOI for b-values $b_{1}$ and $b_{2}$. In our calculations, we determined the ADC using two different b-values: one at 750 or 800 s/mm^2^ and the other close to zero, at 50 s/mm^2^. Additionally, ADC maps were generated from these ADC values.

In the second method, the ADC was calculated for each voxel in the same manner. Then, the mean, minimal, maximal, and median ADC values were calculated.

An internal reference of healthy skin was used to establish ADC values for the non-affected skin, which were later used to assess how much the value of these pathologies differed from normal values in healthy skin.

### 2.5. SNR Estimation

To ensure that the obtained ADC values were correctly computed and not corrupted by an insufficient SNR [35], the SNR was estimated using the following formula:

(2) $$SNR=\frac{S}{\sigma },$$

A 2D region was drawn in a signal-free region outside the body, and the mean signal, $\left|S_{noise}\right|$, of the region was calculated. Then, σ was estimated using the following formula:

(3) $$\sigma =\sqrt{\frac{2}{\pi }}\cdot \left|S_{noise}\right|,$$

For a single-channel coil, this would be a reasonable estimate for the size of the noise floor. However, since we used a multi-channel setup, σ can only be approximated in this manner. The signal, S, (e.g., of the breast’s skin) was estimated using the following formula, considering the correction for Rician distributed noise [36]:

(4) $$S_{tissue,corrected}\approx \sqrt{S_{tissue}^{2}-\sigma^{2}},$$

An SNR value of two was used as a cut-off for images in patient groups with breast skin pathologies. Upon visual inspection, we found this SNR threshold to be appropriate. The SNR of healthy skin was regularly very low, and we used no cut-off for these cases. Therefore, the stated healthy skin ADC values are biased. Nonetheless, we state them because our values are presumably representative of those that would be obtained in a routine clinical workflow.

### 2.6. Statistical Analysis

The mean, maximum, minimum, and median ADCs of five independent groups were tested for normality using the Kolmogorov–Smirnov test. Since these tests indicated non-normality in most cases, the groups were compared using Wilcoxon rank sum tests. Due to the limited number of Paget’s disease cases (*n* = 3) included in this study, assessment of the Paget’s disease samples is only provided as a descriptive analysis. All calculations and statistical analysis were performed using MATLAB (v2020a; MathWorks, Natick, MA, USA). All results with *p*-values < 0.05 were considered statistically significant.

## 3. Results

### 3.1. Demographics

The study population included 88 women, of whom 58 (66%) were control cases with healthy skin (mean age: 51 ± 2 years) and 30 (34%) were cases with skin pathologies (mean age: 59 ± 2 years; 10% (3/30) with PD, 16% (5/30) with IBC, 36% (11/30) with BSI, and 36% (11/30) with SIBC). Further details are provided in Table 2.

**Figure 2:**
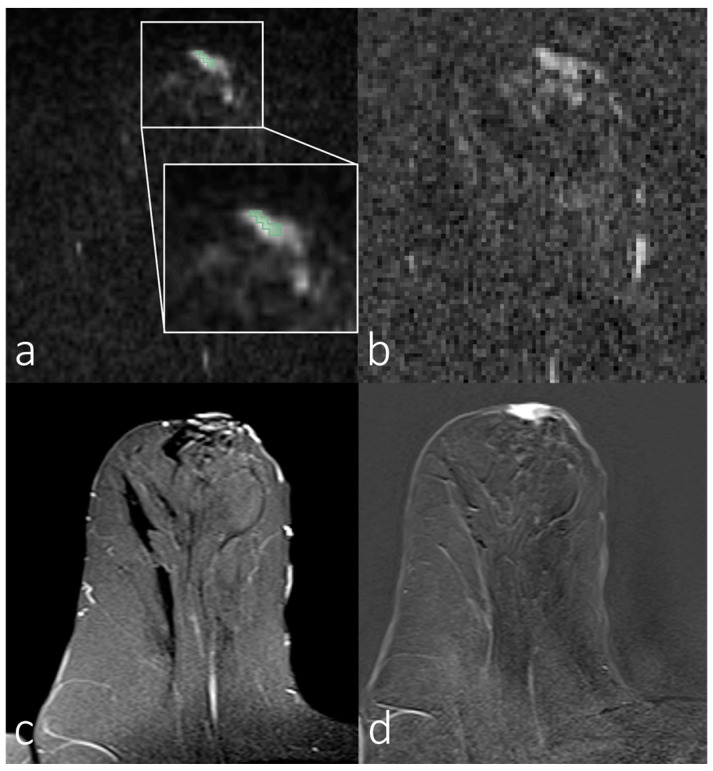
Breast magnetic resonance imaging of a woman with Paget’s disease of the nipple. (**a**) Manual segmentation overlaid on the diffusion-weighted image (b = 800 s/mm^2^), with segmentation highlighted in green; (**b**) apparent diffusion coefficient map; (**c**) T_2_-weighted image with fat saturation; (**d**) T_1_-weighted subtraction image after contrast administration.

**Figure 3:**
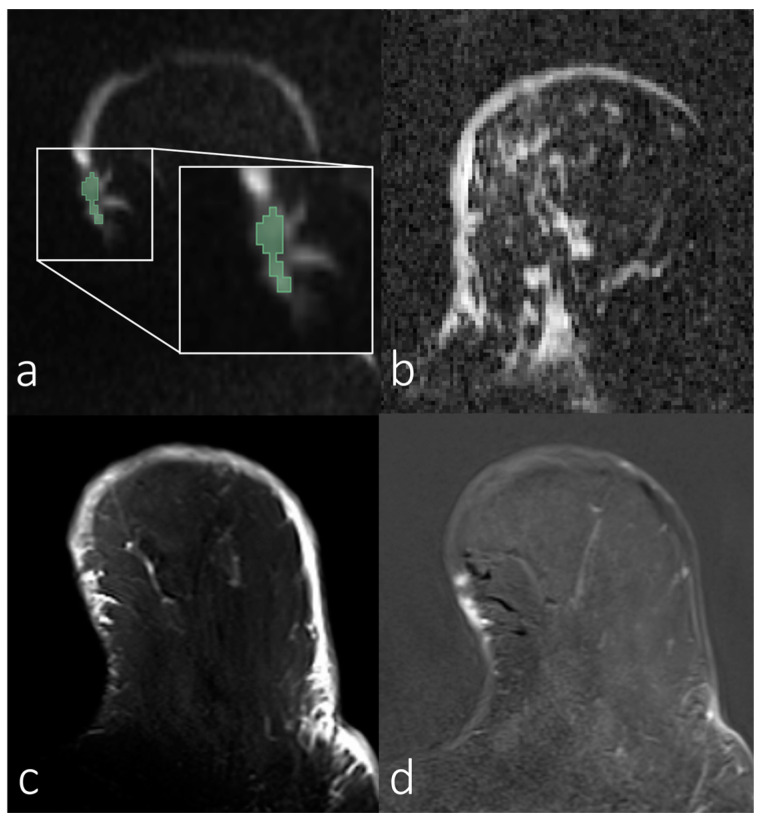
Breast magnetic resonance images of a woman with skin infiltrating mammary carcinoma. (**a**) Manual segmentation overlaid on the diffusion-weighted image (b = 800 s/mm^2^), with segmentation highlighted in green; (**b**) apparent diffusion coefficient map; (**c**) T_2_-weighted image with fat saturation; (**d**) T_1_-weighted subtraction image after contrast administration.

**Figure 4:**
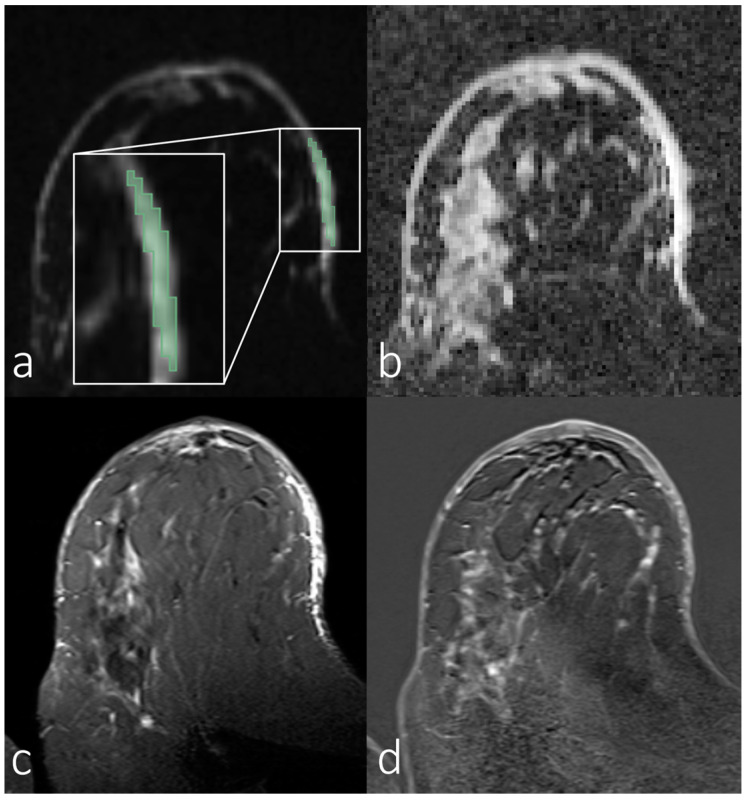
Breast magnetic resonance images of a woman with inflammatory breast carcinoma. (**a**) Manual segmentation overlaid on the diffusion-weighted image (b = 800 s/mm^2^), with segmentation highlighted in green; (**b**) apparent diffusion coefficient map; (**c**) T_2_-weighted image with fat saturation; (**d**) T_1_-weighted subtraction image after contrast administration.

**Figure 5:**
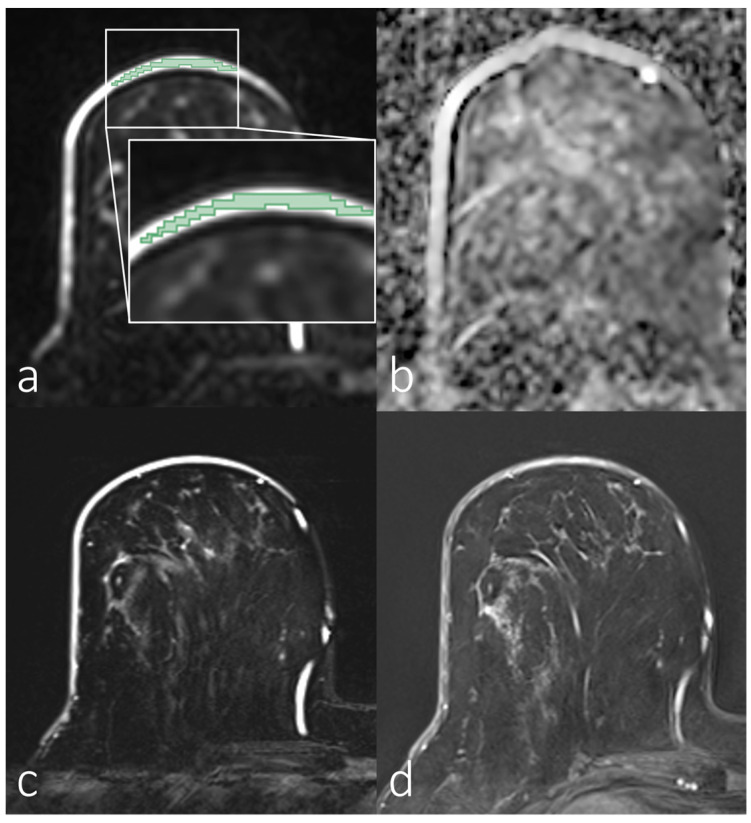
Breast magnetic resonance imaging of a woman with benign skin inflammation (mastitis). (**a**) Manual segmentation overlaid on the diffusion-weighted image (b = 800 s/mm^2^), with segmentation highlighted in green; (**b**) apparent diffusion coefficient map; (**c**) T_2_-weighted image with fat saturation; (**d**) T_1_-weighted subtraction image after contrast administration.

Figure 2, Figure 3, Figure 4 and Figure 5 show representative images of all the skin-involving diseases included in this study (PD, IBC, BSI, and SIBC). The used VOIs are shown as overlays on the diffusion-weighted images (top left subfigures) and were used to infer the ADC values from the ADC maps shown in the top right subfigures. Additionally, T_1_- and T_2_-weighted images are shown. Figure 2 shows a case of PD of the nipple. The lesion appears relatively solitary in the diffusion-weighted image and ADC map, with a fainter appearance than the respective regions of interest in Figure 3, Figure 4 and Figure 5. None of the cases shown in Figure 3, Figure 4 and Figure 5 shows a similar solitary appearance of the region of interest. Figure 3 shows a case of infiltrating mammary carcinoma. It does not appear as singular in the diffusion-weighted image or ADC map as the PD case. Little contrast is visible on the ADC map between the cancer region and the skin region surrounding it. Figure 4 shows an IBC case. Unlike in Figure 3, the bright skin regions in the diffusion-weighted image and ADC map match relatively well with the hyperintense skin region in the contrast-enhanced T_1_-weighted image. Figure 5 shows a BSI case. The skin’s visual appearance is relatively similar to that in Figure 4 in all four subfigures, and differentiation might be difficult.

### 3.2. Analyses of First-Order Statistics Using the ADC

**Figure 6:**
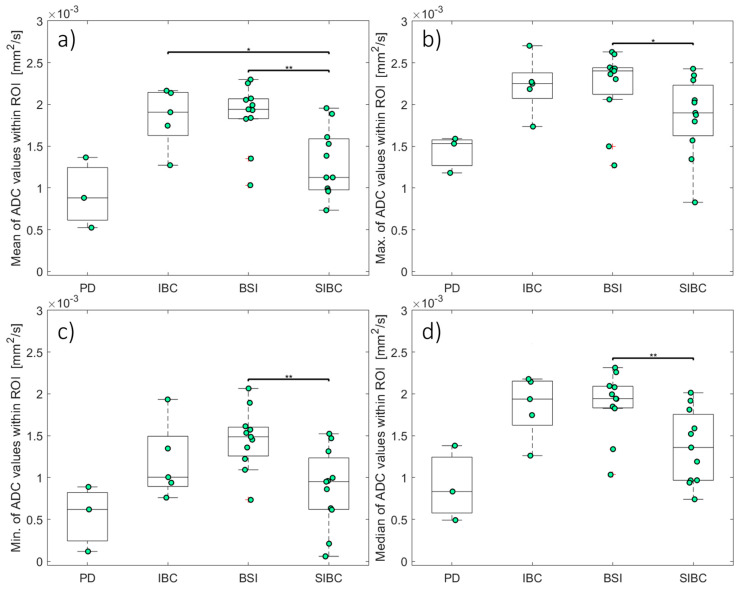
The apparent diffusion coefficients for skin pathologies. Box and whisker plots of the mean (**a**), maximum (**b**), minimum (**c**), and median (**d**) ADCs. The asterisks indicate statistical significance: *, *p* < 0.05; **, *p* < 0.01. ADCs: apparent diffusion coefficients; BSI: benign skin inflammation; IBC: inflammatory breast cancer; PD: Paget’s disease; SIBC: skin infiltration of breast cancer.

Table 3 and Figure 6 present the ADCs for PD, IBC, BSI, SIBC, and healthy skin. The different first-order descriptive statistics (minimum, maximum, mean, and median) of the ADCs in all evaluated groups with skin pathologies differed significantly from those in the healthy control group (*p* < 0.05). Unlike in the groups with skin pathologies, the signal in the healthy skin was often too low to ensure a proper ADC computation. The highest ADCs in the skin pathology groups were in the BSI (1.88 ± 0.11 [1.04–2.30] µm^2^/s) and IBC (1.86 ± 0.17 [1.28–2.18] µm^2^/s) groups, which did not differ significantly (*p* > 0.05). The mean ADC was significantly lower in the SIBC group (1.38 ± 0.13 [0.73–1.98] µm^2^/s) compared to the BSI (*p* = 0.005) and IBC (*p* = 0.027) groups.

**Figure 7:**
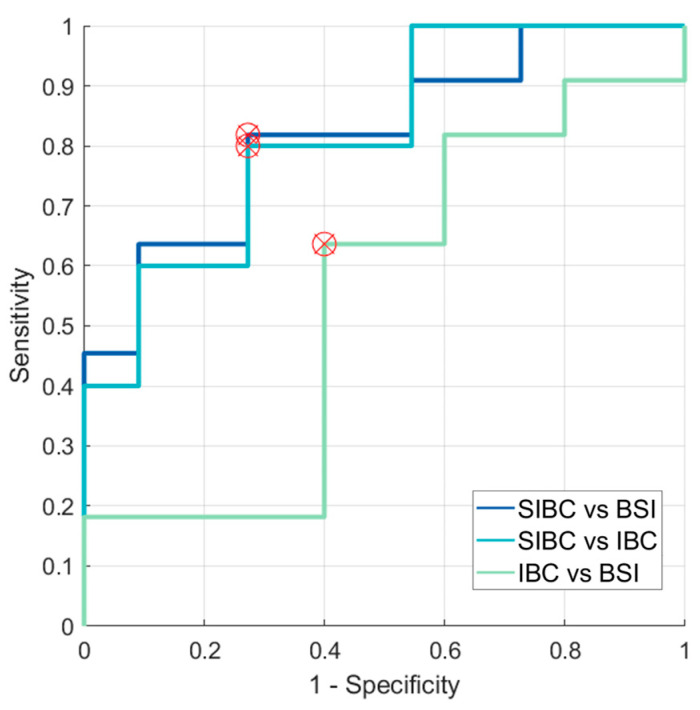
ROC curves highlighting optimal thresholds for ADC-based differentiation between SIBC and BSI, between SIBC and IBC, and between IBC and BSI. ADC: apparent diffusion coefficient; ROC curve: Receiver Operating Characteristic curve; SIBC: skin-involved breast cancer; BSI: benign skin inflammation; IBC: inflammatory breast cancer.

Figure 7 shows the Receiver Operating Characteristic (ROC) for ADCs calculated from the mean signal within the region of interest. The accuracy values, as measured by the area under the curve (AUC), for SIBC and BSI, SIBC and IBC, and IBC and BSI were 0.818, 0.818, and 0.545, respectively. The sensitivities were 81.82%, 80.00%, and 63.64%, respectively. The specificities were 72.73%, 72.73%, and 60.00%.

### 3.3. Evaluation of SNR in the Skin for Assessing ADCs in Skin Pathologies

The results of the quantitative SNR analysis of the VOIs for all cases of different cancer types and healthy skin included in this study are presented in Table 4. The mean and range of the SNRs for diffusion-weighted images with higher diffusion-weighting factors (b-value: 800/750 s/mm^2^) are predictably lower due to the diffusion weighting. Most SNRs were well above the cut-off of two. The SNR in the diffusion-weighted images dropped below the cut-off value in only two cases, which were excluded from this study.

## 4. Discussion

Here, we describe our initial experience deriving quantitative diffusion data for several skin pathologies in breast MRI. Our study demonstrates the technical feasibility of deriving quantitative first-order descriptive statistics of ADCs in skin pathologies and the significant differences between some types of pathologies. However, as further outlined below, we did not find statistically significant differences between the BSI and IBC groups, which might reflect the underlying biological processes of those diseases or challenges associated with the assessment. In contrast, significant differences were observed between the BSI and SIBC groups, potentially indicating usefulness as complementary information in diagnosing such cases.

All pathologies showed ADCs that differed from healthy skin; however, this needs to be thoroughly contextualised. A decrease in an ADC typically indicates reduced water mobility associated with increased “cellularity” [2,3]. Such a decrease can be commonly observed in cancer tissue, which has been suggested to correlate with more cellular restrictions in its architecture than healthy tissue in many malignancies. This limits diffusion and results in a lower diffusion coefficient in abnormal tissue compared to healthy tissue. For normal breast tissue, the reported mean ADC ranges between 1.7 and 2.0 µm^2^/s [37]. However, the ADC may be influenced slightly by the menstrual cycle and breast density and vary between women given and not given hormone replacement therapy [38]. The here-observed ADCs for healthy skin are not within the range of healthy breast tissue because the skin had different water content and cellular structure, resulting in a much lower ADC than in healthy tissue.

Regarding the skin region, Bittounet et al.’s statistically significant findings suggest that the ADC is sensitive to skin ageing due to reduced water mobility in the young compared to the ageing epidermis [39]. According to their study, mean ADCs may vary in different skin layers, such as the epidermis (young [Y]: 2.81 ± 0.25 µm^2^/s; aged [A]: 3.17 ± 0.26 µm^2^/s), outer dermis (Y: 2.33 ± 0.29 µm^2^/s; A: 2.85 ± 0.34 µm^2^/s), or inner dermis (Y: 0.90 ± 0.65 µm^2^/s; A: 1.40 ± 0.60 µm^2^/s), demonstrating a strong gradient towards the subcutaneous fat region. In comparison, the mean ADC of the healthy skin in our study was 0.48 ± 0.02 µm^2^/s, ranging from 0.23 to 0.84, which might reflect the relatively low resolution causing a partial inclusion of fat-suppressed subcutaneous tissue into the voxels. However, the dominant effect is presumably the low signal in higher b-value acquisitions, limiting the SNR and biasing the ADC calculation.

Besides the visual inspection of suspicious skin lesions, various techniques are used to image the skin [40]. These include molecular imaging techniques such as single-photon emission computed tomography or positron electron tomography, as well as anatomical methods like reflectance confocal microscopy, high-frequency ultrasound, optical coherence tomography, near-infrared bioimaging, and magnetic resonance imaging (MRI). Frequently, a combination of these techniques is used. In addition to routine MRI sequences, which primarily facilitate visual assessment, DWI enables quantitative evaluation of tissue. Research studies have demonstrated that this quantitative approach enhances the differentiation between benign and malignant lesions, providing valuable insights into tissue characterisation [41]. Like in many previous studies, the here-observed ADCs indicate that it is possible to distinguish malignant and benign tissue. Many studies have evaluated the diagnostic efficacy of DWI and ADCs in distinguishing between malignant and benign breast lesions and provided ADC ranges or thresholds for differentiating normal tissue, malignant lesions, and benign lesions [31]. The EUSOBI working group provided preliminary ADC ranges for the malignant (0.8–1.3 µm^2^/s) and benign (1.2–2.0 µm^2^/s) to help differentiate them [37]. However, it should be noted that they emphasise that the suggested features, such as ADC ranges, are preliminary, depend on various circumstances, and may evolve with time.

The lesion types examined here were limited to the specific sub-compartment of the skin in the patient’s breast. We found that within the skin, lesions tended to show slightly higher ADCs for malignant pathologies than those reported for malignant and benign tissues outside the skin, which might reflect the interposition of the lesions with skin and the reported higher ADC values for skin [39] than for FGT. It could also be associated with the inflammatory reaction of the skin observed in IBC and other malignant processes in the skin.

In this study, statistical evaluation of the first-order ADC feature “mean” differed significantly between two pairs of groups (IBC and SIBC, BSI and SIBC), while the “median”, “maximum”, and “minimum” features only differed between one pair (see Figure 6). The findings of Bickel et al. [42] suggest that the minimum and mean ADC show the best diagnostic performance. They also suggested that the minimum ADC might represent the most malignant part of the lesion, potentially leading to better differentiation between benign and malignant lesions. Some other studies [43,44] came to similar conclusions and stated that the lowest ADC might be associated with the most malignant part of the carcinoma. This conclusion is consistent with our finding that the comparisons of the “mean” and “minimum” ADCs between the BSI and SIBC groups yielded the lowest *p*-values among all group comparisons. This might be indicative that a significant difference in ADC might be found between these groups with larger sample sizes.

Due to the limited sample size of Paget’s disease (*n* = 3) included in this study, statistical comparison to the other entities was not conducted. However, we acknowledge its relevance and still report descriptive results. The mean ADC in the PD group differed from the BSI group but to a lesser extent than those of the SIBC and IBC groups. In general, Paget’s disease potentially resulted in a lower ADC than the benign skin inflammation findings due to the disease’s increased tissue cellularity and malignant nature. Additionally, IBC and BSI could not be differentiated based solely on the DWI imaging and ADC alone, which might be associated with similar microstructural features underlying the skin thickening and subcutaneous reactive oedema.

This study has several limitations that should be addressed in future research. First, our cohort’s sample size was relatively small, especially for patients with IBC or PD, placing this study more in the range of a methodological study [45]. The inclusion of technically insufficient examinations was inevitable in our retrospective study, which focused on rare cases. It should be considered that the performed statistical tests might have had low statistical power and a limited ability to detect relationships or effects due to the limited sample size and our cohort. Consequently, it does not allow the drawing of any clinical implications.

Second, by evaluating MRI data retrospectively, certain aspects of the examination procedure that could impact ADC calculations were beyond the control of this study. These factors include, i.a., selections of the b-values in clinically used protocols. Previous studies have investigated the optimal b-values for various applications [46], their importance in clinical applications and ADC calculations [47], and ADC thresholds for differentiating between tissues and tumour types that should be defined considering data acquisition parameters [6,48,49].

Another limiting factor beyond the composition of the study cohort is the magnetic field strength of the scanners used. This study evaluated examinations conducted at different magnetic field strengths. Although these examinations were collectively analysed, it was generally observed that the influence of magnetic field strength (B0) on the diffusion metrics was not large (effects in the roughly 20% range), especially when compared to the impact on T_1_. However, the situation appears more subtle, and conflicting reports often exist. No current guideline recommends the use of a certain B0 for achieving better contrast. Regarding the field strengths of 1.5 T and 3 T, Hunsche et al., Brander et al., and Ding et al. found little dependency of human white matter diffusion tensor imaging metrics on B0 [50,51,52], while Huisman et al. and Fushimi et al. found lower mean diffusivity and higher fractional anisotropy values at 3 T with a difference of roughly <10% in size [53,54]. Farhood et al. also showed no significant difference between the 1.5- and 3-T values for the liver, gallbladder, kidneys, and pancreas [55].

Not many studies investigate the effects of echo time (TE) on the quantification of diffusion characteristics. However, Feng et al. showed that there were no significant differences (*p* > 0.05) in the ADC for different TEs in the central zone of the prostate [56]. Qin et al. revealed no correlation between mean diffusivity and TE [57]. Lemke et al. further demonstrated that diffusion coefficients were also not significantly affected in normal intravoxel incoherent motion pancreas imaging at low b-values [58].

Another limitation was that our patient cohort was examined over a relatively long period, including various scanners and examination parameters, necessitating the inclusion of ADC maps calculated from different b-values (66 cases used b-values of 50 and 800 s/mm^2^, and 22 cases used b-values of 50 and 750 s/mm^2^). However, a systematic review and meta-analysis by Ruo-Yang et al. [59] showed that the diagnostic performance of DWI and the ADC in differentiating malignant and benign breast lesions does not differ significantly between units with different field strengths, such as 1.5 T and 3.0 T.

Since the signal in the healthy skin was often too low to ensure a proper ADC computation, the healthy skin ADCs do not represent the correct ADCs due to a limited SNR in the diffusion-weighted images. Nonetheless, we stated them because they should roughly reflect the ADCs that other investigators might derive if a clinical setup is chosen and ADC values are simply measured within an ADC map on the clinical routine PACS. Future studies could consider using a dedicated high-resolution MRI surface coil to achieve potential advancement in this area, greater SNRs, and higher-quality images of every skin layer and skin tumours with increased spatial resolution.

Last but not least, various factors may influence the diffusion of water molecules. Therefore, it is essential to consider the limitations of the mono-exponential decay model as more complex models, such as kurtosis and kurtosis-corrected diffusion models [60,61,62,63], as well as bi-exponential or stretched-exponential models [64,65], which may represent the diffusion in certain tissues differently or more accurately. It is important to note that ADC is a scalar value and does not provide any information about diffusion orientation. To obtain this information, it may be necessary to consider adding other MRI techniques in future studies.

## 5. Conclusions

The skin pathologies of women undergoing breast MRI can be challenging to assess, even with advanced imaging techniques such as DWI. Our results indicate that differences in the quantitative ADC might exist between different breast skin pathologies. IBC and BSI could not be differentiated based on the ADC alone in our cohort. However, supplementing MRI with a quantified approach might partially support the diagnostic assessment of skin pathologies in breast imaging. Further research is necessary to explore the potential applications of DWI in assessing the skin in breast MRI.

## Figures and Tables

**Table 1:** Diffusion-weighted imaging acquisition parameters.

Parameters	Diffusion-Weighted Imaging Sequences
Total scan time	1 min 44 s–4 min 43 s
Magnetic field strength	72% 1.5 T, 28% 3 T
Slices, no.	24–62
Slice thickness (mm)	2.5–5
Spacing between slices (mm)	2.75–6
Repetition time (ms)	4100–9750
Echo time (ms)	54–106
Inversion Time (ms)	0–250
b-values (s/mm^2^), [averages]	50, 400, 800 [2, 3, 4 or 3, 4, 5 or 9, 12, 15]; 50, 750, 1500 [3, 8, 15]
Matrix	128 × 80–220 × 168
Percent phase field of view	48.98–76.36
Pixel spacing (mm)	1.37–2.50
Pixel bandwidth (Hz/Px)	1263–2300

**Table 2:** Descriptive statistics of the study cohort, scan parameters, and segmentations.

Type	No.	Mean Nr of Voxels in VOI ^1^	Magnetic Field Strength		b-Values (s/mm^2^)		Occurrence and VOI Placement		Age (Years)
			1.5 T	3 T	750	800	Right Breast	Left Breast	
Paget’s disease of the nipple	3	35	1	2	1	2	1	2	54 ± 8
Inflammatory breast cancer	5	258	1	4	3	2	2	3	55 ± 5
Benign skin inflammation or enhancement	11	197	5	6	5	6	3	8	59 ± 4
Skin infiltration breast cancer	11	46	4	7	6	5	7	4	61 ± 5
Healthy skin	58	29	52	6	6	52	30	28	51 ± 2

^1^ VOI: volume of interest.

**Table 3:** Overview of the investigated apparent diffusion coefficients in the breast skin (means ± standard deviation).

	Median ADC ^1^ Value (µm^2^/s)	Max. ADC ^1^ Value (µm^2^/s)	Min. ADC ^1^ Value (µm^2^/s)	ADC ^1^ of Mean Signal within VOI ^2^ (µm^2^/s)	Mean of ADC ^1^ Values within VOI ^2^ (µm^2^/s)
Paget’s disease of the nipple	0.90 ± 0.26 (0.49–1.38)	1.43 ± 0.13 (1.18–1.59)	0.54 ± 0.23 (0.12–0.89)	0.95 ± 0.23 (0.59–1.37)	0.92 ± 0.24 (0.52–1.36)
Inflammatory breast cancer	1.85 ± 0.17 (1.26–2.18)	2.23 ± 0.15 (1.74–2.71)	1.2 ± 0.21 (0.76–1.93)	1.86 ± 0.17 (1.28–2.18)	1.84 ± 0.16 (1.27–2.16)
Benign skin inflammation or enhancement	1.88 ± 0.11 (1.03–2.31)	2.22 ± 0.13 (1.27–2.63)	1.46 ± 0.11 (0.73–2.06)	1.88 ± 0.11 (1.04–2.3)	1.87 ± 0.11 (1.03–2.3)
Skin infiltration breast cancer	1.36 ± 0.13 (0.74–2.01)	1.86 ± 0.14 (0.83–2.43)	0.87 ± 0.14 (0.06–1.52)	1.38 ± 0.13 (0.73–1.98)	1.37 ± 0.13 (0.73–1.95)
Healthy skin	0.48 ± 0.02 (0.22–0.84)	0.86 ± 0.03 (0.42–1.47)	0.18 ± 0.02 (0.01–0.63)	0.49 ± 0.02 (0.23–0.85)	0.48 ± 0.02 (0.23–0.84)

^1^ ADC: apparent diffusion coefficient; ^2^ VOI: volume of interest.

**Table 4:** Quantitative results of signal-to-noise ratios of the diffusion-weighted magnetic resonance images for all evaluated groups.

Type	Signal-to-Noise Ratio	
	50 s/mm^2^	800/750 s/mm^2^
Paget’s disease of the nipple	36 ± 27 (4.7–54)	14 ± 10 (2.4–22)
Inflammatory breast cancer	239 ± 218 (24–588)	59 ± 46 (4.6–117)
Benign skin inflammation or enhancement	199 ± 324 (23–870)	56 ± 101 (5.1–338)
Skin infiltration breast cancer	97 ± 113 (9.2–370)	37 ± 49 (2.9–168)
Healthy skin	3.09 ± 3.71 (0.95–20)	2.04 ± 2.97 (0.6–16)

## Data Availability

The datasets generated and analysed during the current study are not publicly available due to the general data protection regulation (GDPR) but are available from the corresponding authors upon reasonable request.

## Supplementary Materials

The following supporting information can be downloaded at: https://www.mdpi.com/article/10.3390/diagnostics14090934/s1. The supplemental digital content contains the keyword list referred to in this document.
