# Supplementary material for: Diffusion-Weighted Imaging for Skin Pathologies of the Breast—A Feasibility Study
Source: Diagnostics (Basel). 2024 Apr 29;14(9):934. doi: 10.3390/diagnostics14090934 (PMC11083106; doi:10.3390/diagnostics14090934)
Supplement: Supplementary file 1 [file diagnostics-14-00934-s001.zip › diagnostics-2930091-supplementary.pdf]

Supplemental Digital Content S1. Keyword list (text document)

The identification of patients eligible for inclusion in the study involved a keyword search in our in-house structured database. The keyword list is provided in this text document as supplemental material to the manuscript "Diffusion-Weighted Imaging for Skin Pathologies of the Breast – A Feasibility Study". Since the medical reports were in German language, keywords were chosen mostly in the German language as well.

- Infiltration
- Pagetoid
- Paget
- Morbus Paget
- inflammatorische
- Cutis Infiltration
- Cutis
- Cutisverdickung
- Verdickte Cutis
- Haut
- Skin
- Hautbiopsie
- Hautverdickung
- Hautinfiltration
- Entzündlich
- Reaktive Veränderungen
- Benigne
- Rötung
- Mastitis
- Kutane Metastase
- Kutane Infiltration
- Kutaner Infiltration
- Kutane Beteiligung
- Intramammäre
- Kutane Läsionen
- Brustdrüsengewebe
- Restdrüsenparenchym
- Folliculitis
- Inf.
- Inflam.
- Inflamm.
- Inflamm. MaCa
- Ödematöse
